# Effects of flavonoids from *Martynia annua* and *Tephrosia purpurea* on cutaneous wound healing

**Published:** 2016

**Authors:** Santram Lodhi, Avijeet Jain, Alok Pal Jain, Rajesh Singh Pawar, Abhay Kumar Singhai

**Affiliations:** 1 *Department of Pharmaceutical Sciences, Dr. H. S. Gour University, Sagar, Madhya Pradesh, India-470003*; 2 *Department of Pharmacy, RKDF University, Bhopal, Madhya Pradesh, India-462033*; 3 *Guru Ramdas Khalsa Institute of Science & Technology, Pharmacy, Kukrikheda, Barela, Jabalpur (M.P.), India*; 4 *Department of Pharmacognosy, VNS Institute of Pharmacy, Bhopal, Madhya Pradesh, India*

**Keywords:** *Martynia annua*, *Tephrosia purpurea*, *Povidone Iodine ointment*, *Burn wound*, *Luteolin*, *Dead-space wound*

## Abstract

**Objective:**

*Martynia annua* L. (*M. annua*), (Martyniaccae) has been traditionally used in the treatment of epilepsy, sore throat and inflammatory disorders. The leaf paste is used topically on Tuberculosis of the lymphatic glands and wounds of domestic animals. *Tephrosia purpurea *(*T. purpurea*), (Fabaceae) has been used traditionally as a remedy for asthma, gonorrhea, rheumatism and ulcers. This study aimed to evaluate the potential wound healing effects of different fractions ofethanol extract of *M. annua* leaves and aerial parts of *T. purpurea*.

**Materials and Methods:**

Methanol fraction of *M. annua* (MAF-C) and ethyl acetate fraction of *T. purpurea* (TPF-A) were evaluated for healing potential in dead-space and burn wound models. An ointment (5% w/w) of MAF-C and TPF-A, pongamol (0.2 and 0.5% w/w) and luteolin (0.2 and 0.5% w/w) was applied topically twice a day. The effects were compared with Povidone Iodine ointment with respect to protein, collagen content, enzymatic assay and histopathological finding of granuloma tissues.

**Results:**

Ethanol extracts of *M. annua* and *T. purpurea*were exhibited total flavonoid contents of 126.2 ± 4.69 and 171.6 ± 6.38 mg (quercetin equivalent), respectively. HPLC fingerprinting confirmed the presence of luteolin in* M. annua *and quercetin in *T. purpurea*. TPF-A and MAF-C ointments (5% w/w) significantly increases the hydroxyproline and protein contents. Luteolin and pongamol ointments were also found to be effective in both wound models.

**Conclusion:**

Our findings suggested that 5% w/w ointment of TPF-A and MAF-C fractions were more effective than isolated flavonoids in wound healing which may be due to synergistic interactions between the flavonoids and other constituents.

## Introduction

Wound healing is a complex physiological process that requires a series of steps, each with several factors up to completion. The sequential phases of healing process are inflammation, proliferation and migration of connective tissue, production of extracellular matrix including collagen synthesis, epithelial cells migration and proliferation leading to neovascularization of wounded tissue. The inflammatory phase includes changes in capillary permeability, transudation and cellular migration leading to second proliferation phase, in which proliferation of fibroblasts, endothelial cells occurs in injured areas. The last is remodeling phase in which cells production is balanced by cell death, collagen production by degradation and absorption and capillary formation by capillary obliteration (Shukla et al., 1999 ▶; Gou and Dipietro, 1980 ▶).

*Martynia annua *L. (Martyniaccae family), is an annual glandular hairy herb which is commonly known as Bichchhu. It is used in epilepsy and is locally applied to the Tuberculosis of lymphatic glands of camel’s neck. The juice of leaves is used as a gargle for sore throat, fruit in inflammatory ailments and leaf paste is topically used for wounds in domestic animals (Khare, 2007 ▶). The root extract exhibited fungicide activity against *Acaulospora scrobiculata, Sclerocystis sinuosa *(Kamble et al., 2012 ▶). Phytochemical studies on *M. annua* revealed the presence of glycosides, flavonoids, tannins, carbohydrates, phenols, and anthocyanins in leaves (Lodhi and Singhai, 2013 ▶). Seeds have been reported to have linoleic acid, arachidic acid, pelargonidin-3-5-diglucoside, palmitic acid, apigenin and apigenin-7-*O*-glucuronide (Gunasegaran and Vidya, 1992 ▶; Rastogi and Melhotra, 1993 ▶). Flowers contain cyanidin-3-galactoside whilst *p*-hydroxy benzoic acid and sinapic acid are found in leaves and gentisic acid is present in fruits (Mali et al., 2002 ▶). The anti-inflammatory activity of ethanol extract of *Martynia diandra *Glox was investigated in acute and sub-acute inflammatory experimental models (Chatpalliwar et al., 2002 ▶).

*Tephrosia purpurea *L. (Fabaceae), an important indigenous medicinal plant of Indian Medicinal System is commonly known as Sarpunkha. It is the active component of Tefroli and Yakrifit herbal preparations used for liver disorders. In clinical trials, Tefroli tablet is well tolerated and no side effect was noticed in any of 32 cases during the treatment of viral hepatitis. Yakrifit is a liver tonic of *T. purpurea *which is found clinically effective for veterinary uses (Sankaran, 1980 ▶; Kumar et al., 1997 ▶). In Ayurveda, *T. purpurea* is used as a remedy for asthma, diarrhoea, gonorrhoea, allergic and inflammatory conditions such as rheumatism, ulcers and urinary disorders (Kirtikar and Basu, 1956 ▶; Despande et al., 2003 ▶).The aqueous extract of seeds has shown insecticidal and insect repellant properties and significant *in vivo* hypoglycemic activity in diabetic rabbits (Rahman et al., 1985 ▶). The plant is also effective in bilious febrile attacks, boils, pimples, bleeding piles and obstruction of liver and spleen. The roots are reported to be effective in the treatment of leprous wounds and its juice is effective against the eruption on skin. Alcoholic extract of pods is effective inthe treatment of pain and inflammation, while their decoction is used against vomiting (Anonymous, 1976 ▶). The phytochemical investigations on aerial parts of *T.purpurea,* revealed the presence of fatty acids, flavonoids, steroids, and amino acids (Lodhi et al., 2013 ▶). Leaves contain 1-3% of rutin, β-sitosterol, quercetin, lupeol and seeds contain purpuritenin, purpureamethide, pongamol, karanjin, lanceolatin B (Sinha et al., 1982 ▶) and a number of fatty acids e.g. linoleic, oleic, palmitic, stearic and linolenic acid (Zafar et al., 2004 ▶). The roots contain tephrosin, rotenone, degulin and elliptone (Gupta et al., 1980 ▶). According to Ayurveda, *T. purpurea* has been also known as “*Sarwa wranvishapaka”* which means that it has healing property for all types of wounds. In a preliminary study, we reported that the ethanol extract of aerial parts of *T. purpurea *was effective in wound healing (Lodhi et al., 2006 ▶). The present study was aimed to evaluate wound healing potential of different fractions and flavonoid constituents of ethanol extract of *M. annua *leaves and aerial parts of *T. purpurea *using dead-space and burn wounds models.

## Materials and Methods

**Plant material**

The leaves of *M. annua *and aerial parts of the *T. purpurea* were collected from the campus of University during the summer (2009). The plants were identified as *M. annua *L. (Bot/Her/267) and *T. purpurea *(Bot/Her/554) in the department of Botany, Dr. Hari Singh Gour University Sagar, M.P., India. The plant materials were dried in shade, powdered and stored in well-closed containers.

**Chemicals and reagents**

Methanol, sodium nitrate, hydrochloric acid, aluminum chloride, sodium hydroxide, toluene, chloroform, acetone, benzene, sodium tartrate, copper sulphate and sodium carbonate, perchloric acid and ethyl acetate, were purchased from Ranbaxy Fine Chemicals Ltd., Thane, India. All chemicals were of analytical grade. Folin-Ciocalteau reagent, Ehlrich reagent, quercetin, luteolin, superoxide dismutase (SOD), catalse (CAT) and reduced glutathione (GSH) were purchased from Sigma Chemical Co. (USA). Hydroxyproline and Trichloracetic acid (TCA) were purchased from Himedia Laboratories Ltd. (Mumbai, India).

**Extraction and** **phytochemical screening**

The powdered plant materials of *M. annua* leaves (2 kg) and aerial parts of *T. purpurea* (2 kg) were defatted with petroleum ether (60-80°C) and extracted with ethyl alcohol (95%) up to complete exhaustion. The ethanol extracts of both plants were subjected to phytochemical screening to detect the presence of different chemical constituents.

**Formulation preparation of fractions and isolated flavonoids**

In the previous study (Lodhi and Singhai, 2013 ▶; Lodhi et al., 2013 ▶), we reported that methanol soluble fraction from *M. annua* (MAF-C) and ethyl acetate fraction from *T. purpurea *(TPF-A) were found to be the most active fractions for wound healing. Both fractions (MAF-C and TPF-A) were formulated as5% w/w ointment with simple ointment base separately by using fusion method (Jain and Sharma, 1998 ▶). Pongamol (0.2 and 0.5% w/w) and luteolin (0.2 and 0.5% w/w) were taken for ointment preparation using simple ointment base British Pharmacopoeia (Anonymous, 1953 ▶). All formulations were subjected to dermal irritation study on rat skin. Prepared ointments of active fractions, pongamol (PONG) and luteolin (LUT) were evaluated for cutaneous wound healing.

**Total flavonoid content**

Total flavonoid content was determined by aluminum chloride colorimetric assay (Park et al., 2008 ▶). A test tube containing 0.3 ml of extracts, 3.4 ml of 30% methanol, 0.15 ml of NaNO_2_ (0.5 M) and 0.15 ml of AlCl_3_.6H_2_O (0.3 M) was shaken up to complete mixing. One ml of NaOH (1 M) was added after 5 min and the tube was shaken well. Then, the absorbance was measured at 510 nm. The standard curve of quercetin was made and the total flavonoids content was expressed as milligrams of quercetin equivalents per 100 gm of dried extract.

**HPTLC fingerprinting**

The chromatographic study was carried out to identify and detect phytoconstituents in the fractions. High Performance Thin Layer Chromatography (HPTLC) of TPF-A and MAF-C fractions was done to detect the presence of quercetin and luteolin, respectively. The HPTLC of different fractions were carried out on a pre-coated silica gel plate (0.2 mm, Merck 60 F-254, Germany) as the stationary phase using toluene: chloroform: acetone (40:25:35) as a mobile phase for MAF-C. The mobile phase for TPF-A fraction was benzene: chloroform: methanol (11:9:2). Dried fractions were dissolved in methanol (10 mg/ml) and filtered the solutions. The samples (10 μl) of fractions and standards were spotted in the form of bands with a 100 μl Hamilton syringe on pre-coated silica gel aluminum plate (10 cm ×10 cm) with the help of Linomat 5 applicator. The applicator was attached to the computer system, which was programmed through WIN CATS software. The linear ascending development was carried out in a 20 cm × 10 cm twin trough glass chamber saturated with the mobile phase. The developed plate was dried by hot air to evaporate solvents and placed in a UV chamber set at 254 nm. Spots were scanned in densitometer (CAMAG Scanner 3) under UV light at 254 nm. The R_f_ values and fingerprint data were recorded by WIN CATS software. The operating conditions were as follows: syringe delivery speed:10 s/µl; injection volume:10 µl; band width:6 mm; space between two bands:15 mm; start position:15 mm; lamp:D2 and distance from bottom of plate:15 mm. 

**Dermal irritation study**

A primary skin irritation test was conducted on rat’s skin to determine the irritant effects of extract ointment after a single topical application. Six healthy young adult albino rats of either sex were allowed to have free access to commercial pellets diet and water *ad libitum*. Animals were acclimated to laboratories conditions for a period of 9 days prior to initiation of the experiment. Animal room temperature was maintained at 19-24°C. 

Before starting the dosing, the animals were examined for health and their skin was checked for any abnormalities. No preexisting skin irritation was observed. The extract ointment (5-10 g) was applied to 6 cm^2^ intact area on each animal and the animals were caged. Four hr after exposure to extract ointment, the test sites were gently cleaned of any residual substance. Each test dose was scored according to Draize Scoring System at 1, 24, 48 and 72 hr after removal of test ointment (Draize et al., 1944).The degree of irritancy was obtained by calculating the primary dermal irritation index (PDII).

$\mathrm{PDII}=\frac{\mathrm{PDI for 1,24},\mathrm{48 and 72h}}{4}$

Primary dermal irritation (*α*) = Average erythema + Average edema

**Scoring system**

 0: No erythema and edema; 1: Very slight edema and erythema; 2: Slight edema and erythema (edges of area well defined); 3: Moderate severe edema and erythema; 4: Total possible erythema and edema score.

**Wound healing activity**

*Animals*

Wistar albino rats (150-200 g) of either sex were selected for study. Animals were procured from Defense Research and Development Establishment (DRDE), Gwalior (M.P.). They were housed individually in a well-ventilated, controlled-temperature (26 ± 2°C) animal room for seven days before experimentation. The animals were given standard commercial pellet rodent diet (Hindustan Lever Pvt Ltd, Bangalore, India) and water *ad libitum*. The study protocol were reviewed and approved by the Institutional Animal Ethics Committee (Reg. No. 379/01/ab/CPCSEA). Each group had six animals. The group I was referred as control group, received ointment base, while Groups II-VII denoted as treatment groups that received topically MAF-A, TPF-A, LUT ointment (0.2% and 0.5% w/w), PONG (0.2% and 0.5% w/w) ointment, respectively. The Group VIII received 5% w/w Povidone Iodine Ointment USP (Zenith Drugs Pvt. Ltd, India) and was used as reference group. Ointments were applied twice daily and healing property was assessed in terms of physical, biochemical parameters and histopathological changes. Silver Sulphadiazine (Flammazine^TM^, Smith & Nephew Healthcare Limited, Hull, Canada) ointment was used as a reference in burn wound model.

*Dead space wound model*

Study of granuloma tissue formation in dead-space wound model was done for the determination of dry granulation weight and estimation of biochemical parameters. Animals were anaesthetized by light ether and wound was made by implantation of a polypropylene tube (2.0 × 0.5), on either side, in the lumber region on the dorsal surface of animals. On the 9^th^ post-wounding day, granuloma tissue formed on implanted tube was dissected out carefully. Granuloma tissue from tubes was collected, dried (60°C), weighed and stored in 10% formalin for the estimation of biochemical parameters (Patil et al., 2001 ▶; Shirwaikar et al., 2003 ▶).

*Burn wound model*

The rats were anesthetized by light ether. Hair on the back of each rat was removed with an electric clipper. After hair removal, the dorsal skin surface was exposed to 95°C water for 10 sec through a template designed to produce a full thickness burn injury (Nakae and Inaba, 2000 ▶).The prepared formulations and reference ointment were applied daily up to complete healing. After day 18, the tissue samples were collected for biochemical estimation as well as histopathological study. One part of granulation tissues was collected in phosphate-buffered saline for the estimation of GSH, CAT and SOD levels.

*Protein estimation*

On the post-wounding days, the protein content of skin tissues were determined by method of Lowry et al. (1951) ▶. The tissue lysate was treated with a mixture of sodium tartrate, sodium carbonate and copper sulphate. After 10 min, the mixture was treated with Folin-Ciocalteau reagent that resulted in a bluish color in 20-30 min. The absorbance was read at 650 nm using a UV spectrophotometer (Agilent Technologies, USA).

*Collagen estimation*

Wound tissues were analyzed for hydroxyproline content, which is an essential component of collagen. Tissues samples were dried at 60-70°C up to constant weight in a hot air oven and hydrolyzed by 6 N HCl at 130°C for 4 hr in covered tubes. The hydrolysate solution was neutralized and then, subjected to Chloramine-T oxidation for 20 min (Woessner, 1961 ▶). The reaction was completed by the addition of 0.4 M perchloric acid and brownish to pink color was developed with Ehlrich reagent at 60°C. The sample was read at 557 nm using UV spectrophotometer.

**Enzymatic and non-enzymatic antioxidant assay**

The granuloma tissues were collected from all treated rats and analyzed by antioxidant assays. The tissues were homogenized in phosphate buffer at pH 7.0 and centrifuged under cold condition. The clear supernatant was collected to assay the antioxidants levels. Catalase was estimated following the breakdown of hydrogen peroxide (Beers and Sizer, 1952 ▶). SOD was assayed (Misra and Fridovich, 1972 ▶) based upon the inhibition of epinephrine autoxidation by the enzyme. GSH level was determined by method of Moron et al. (1979) ▶. Homogenates were immediately precipitated with 25% TCA (0.1 ml) and the precipitate was separated after centrifugation. The solution containing free-SH groups was assayed by the addition of 0.6 mM DTNB (2 ml) and 0.9 ml of 0.2 mM sodium phosphate buffer (pH 8.0) to the supernatant (0.1 ml) and the absorbance was read at 412 nm using UV spectrophotometer. 

**Histopathological Study**

Animals were anaesthetized before taking skin samples using diethyl ether. The wound tissue specimen from all treated groups were collected and stored in 10% formalin. Then, 6 μm thick sections were cut and stained with haematoxylin and eosin (McManus and Mowry, 1965 ▶). The histopathologic criteria were used in each animal for: epithelial proliferation, newly formed capillaries, granuloma tissue formation and organization. Sections were qualitatively assessed for fibroblast proliferation, collagen maturation, epithelialization and angiogenesis under light microscope. 

**Statistical analysis**

Pharmacological data were represented as mean ± S.D. for six rats and data were evaluated using the Tukey test. Values of p<0.01 were considered to be statistically significant.

## Results

**Phytochemical studies**

The phytochemical analysis of ethanol extract (4.7% w/w) of *M. annua* revealed the presence of terpenoids, flavonoids, alkaloids, carbohydrates, proteins, tannins, and glycosides. Ethyl alcohol extract (9.5% w/w) of *T. purpurea *had positive results for the presence of terpenoids, flavonoids, carbohydrates, proteins, saponins and amino acids. The total flavonoid contents were found to be 126.2 ± 4.69 and 171.6 ± 6.38 mg (quercetin equivalent) per 100 g ethanol extract for *M. annua* and *T. purpurea*, respectively.

**Figure 1:**
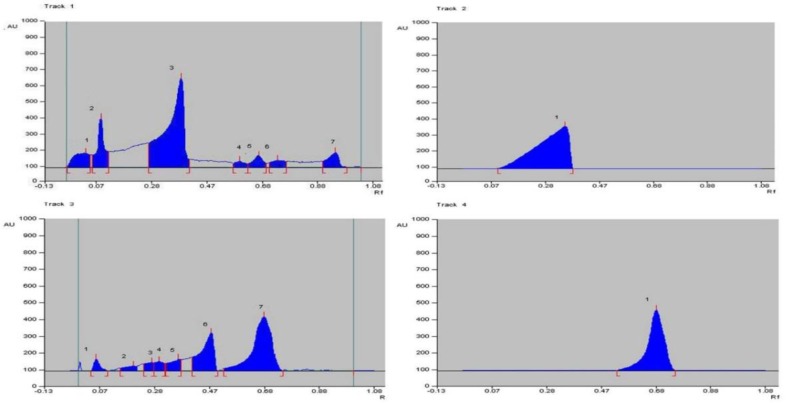
HPTLC fingerprint of different fractions of *M. annua* and *T. purpurea*; 1: TPF-A; 2: Quercetin; 3: MAF-C; 4: Luteolin

HPTLC fingerprinting of different fractions of *M. annua and T. purpurea *revealed several peaks (Figure 1) at 366 nm (Table 1). The standard compounds, quercetin and luteolin gave single spot at R_f_ 0.38 and 0.69, respectively. The first track of TPF-A revealed seven spots with R_f_ values in the range of 0.05 to 0.95. Track third of MAF-C fraction showed seven peaks with R_f_ values in the range of 0.05 to 0.69. 

**Table 1:** HPTLC fingerprint of different fractions of *M. annua* and *T. purpurea*

**Sample**	**Peak**	**Max. R**_f_	**Area**
**TPF-A**	1	0.05	1254.0
	2	0.12	9246.3
	3	0.38	21572.4
	4	0.58	428.1
	5	0.63	2543.5
	6	0.74	2317.8
	7	0.95	985.4
**Quercetin**	1	0.38	12415.5
**MAF-C**	1	0.05	642.7
	2	0.21	533.2
	3	0.26	2451.4
	4	0.31	2538.7
	5	0.36	2749.8
	6	0.49	10247.2
	7	0.69	13876.8
**Luteolin**	1	0.69	14638.4

TPF-A: ethyl acetate fraction of *T. purpurea*; MAF-C: methanol soluble fraction of *M. annua.*

**Dermal irritation study**

Results of irritation study showed that at 1, 24, 48 and 72 hr after the test sample application, no erythema and edema was found at all six treated sites. The total primary dermal irritation and index were also found to be zero. 

**Wound healing activity**

*Dead space wound model*

Over the 9-day treatment period, the protein content of TPF-A and MAF-C -treated groups were found to be 58.54 ± 2.45 and 56.24 ± 1.81, respectively. Protein content was significantly (p<0.01) increased when compared to control group (32.63 ± 1.15). The results were comparable with the reference ointment group (62.89 ± 2.10). Treatment with PONG (0.5% w/w) and LUT (0.5% w/w) ointments were significantly (p<0.01) increased the protein content (41.64 ± 1.62 and 44.72 ± 1.60, respectively) as compared to the control group. No significant increase was observed in the group treated with0.2% w/w of PONG and LUT ointment. Treatment with TPF-A and MAF-C caused a significant (p<0.01) difference in the hydroxyproline content of the wound tissue compared to the control group (Table 2). The wounds treated with 0.5% w/w PONG and 0.5% w/w LUT ointments had less difference in hydroxyproline content as compared to the control group. The group treated with 0.2% w/w of PONG and LUT ointments was found approximately similar to the control group. The granuloma weight in the group treated with TPF-A (52.52 ± 2.14) and MAF-C (56.26 ± 1.52) was found significantly (p<0.01) increased as compared to the control group. The results were found comparable with the reference group. The granuloma weight of PONG (0.2% w/w) and LUT (0.2% w/w)-treated groups were not significantly higher than control group (30.64 ± 1.02) (Table 2).

**Table 2:** Effects of ointment formulations of *M. annua* and *T. purpurea* on various biochemical parameters of tissues with dead-space wound in rats

**Groups**	**Hydroxyproline** **(mg/g tissue)**	**Protein content** **(mg/g tissue)**	**Granuloma dry** **weight (mg)**
**Control (Base)**	17.57 ± 0.64	32.63 ± 1.15	30.64 ± 1.02
**TPF-A Ointment (5% w/w)**	36.82 ± 1.21*	58.54 ± 2.45*	52.52 ± 2.14*
**MAF-C Ointment (5% w/w)**	41.62 ± 2.03*	56.24 ± 1.81*	56.26 ± 1.52*
**PONG Ointment (0.2% w/w)**	19.69 ± 0.43	31.18 ± 1.54	31.77 ± 1.18
**PONG Ointment (0.5% w/w)**	29.28 ± 1.81*	41.64 ± 1.62*	47.28 ± 1.63*
**LUT Ointment (0.2% w/w)**	20.17 ± 0.97	34.89 ± 1.27	33.16 ± 1.20
**LUT Ointment (0.5% w/w)**	28.61 ± 1.25*	44.72 ± 1.60*	45.30 ± 1.84*
**Povidone Iodine Ointment (5% w/w)**	43.26 ± 1.79*	62.89 ± 2.10*	57.85 ± 1.64*

Values represent Mean ± S.D (n = 6).

* p<0.01, when each treated group was compared with control and reference group. TPF-A: ethyl acetate fraction of *T. purpurea*; MAF-C: methanol soluble fraction of *M. annua*; PONG: Pongamol; LUT: Luteolin

The levels of antioxidant substances in skin tissues are given in Table 3. Significant decrease in antioxidants level was observed in control group as well as in 0.2% w/w PONG and 0.2% w/w LUT ointment-treated animals when compared with reference group and other treated groups. Slight increases in SOD, CAT and GSH were found in groups treated with0.5% w/w PONG and LUT ointment. The antioxidants (SOD and GSH) levels in TPF-A, MAF-C and reference ointment-treated animals did not show significant differences on day 9 as compared to control group (Table 3).

**Burn wound model**

Hydroxyproline content in the wound tissues are shown in Table 4. The hydroxyproline contents in animals treated with TPF-A (63.54 ± 2.40) and MAF-C (71.65 ± 2.69) were found to be significantly (p<0.01) greater than the control (41.27 ± 1.57) group. The increase in hydroxyproline content may be attributed to an increase in collagen synthesis or an increase in proliferation of fibroblast cells which synthesize collagens. The wounds treated with 0.5% w/w PONG (50.95 ± 1.20) and LUT (52.10 ± 1.92) ointment showed less significant differences in hydroxyproline content as compared to the control group. Over the post-wounding days, the protein contents of wound tissues treated with TPF-A and MAF-C were 72.40 ± 2.14 and 85.57 ± 3.64, respectively, which were significantly (p<0.01)greater than that of control group. Treatment with 0.5% w/w PONG and LUT ointment, exhibited less increase in the protein content than the control group. No significant increase was found in group treated with 0.2% w/w PONG and LUT ointment.

**Table 3:** Effects of ointment formulations from *M. annua* and *T. purpurea* on antioxidants level of tissues with dead-space wound in rats

**Groups**	**Enzymatic and non-enzymatic assay**		
	**SOD (μg/50 mg tissue)**	**CAT (μmol/50 mg tissue)**	**GSH (μmol/50 mg tissue)**
**Control (Base)**	11.23 ± 0.42	10.71 ± 0.55	12.38 ± 0.38
**TPF-A Ointment (5% w/w)**	26.11 ± 1.25*	34.91 ± 1.13*	26.17 ± 1.14*
**MAF-C Ointment (5% w/w)**	28.52 ± 1.01*	41.22 ± 1.46*	25.48 ± 1.21*
**PONG Ointment (0.2% w/w)**	12.07 ± 0.57	13.28 ± 0.64	10.25 ± 0.28
**PONG Ointment(0.5% w/w)**	20.24 ± 1.02*	25.19 ± 1.20*	22.46 ± 1.12*
**LUT Ointment (0.2% w/w)**	14.10 ± 0.28	9.08 ± 0.48	14.09 ± 0.58
**LUT Ointment (0.5% w/w)**	21.37 ± 1.10*	27.39 ± 1.34*	23.12 ± 1.14*
**Povidone Iodine ointment (5% w/w)**	30.21 ± 1.48*	39.88 ± 1.79*	28.61 ± 1.21*

Values represents Mean ± S.D. (n = 6).

* p<0.01, when each treated group was compared with control and reference group

**Table 4:** Effects of ointment formulations from *M. annua* and *T. purpurea* on hydroxyproline and protein content of tissues with burn wound in rats

**Groups**	**Hydroxyproline content (mg/g tissue)**	**Protein content (mg/g tissue)**
**Control (Base)**	41.27 ± 1.57	50.62 ± 1.58
**TPF-A Ointment (5% w/w)**	63.54 ± 2.40*	72.40 ± 2.14*
**MAF-C Ointment (5% w/w)**	71.65 ± 2.69*	85.57 ± 3.64*
**PONG Ointment (0.2% w/w)**	45.41 ± 1.27	55.43 ± 1.86
**PONG Ointment(0.5% w/w)**	50.95 ± 1.20*	61.71 ± 2.25*
**LUT Ointment (0.2% w/w)**	46.28 ± 1.34	52.38 ± 1.68
**LUT Ointment (0.5% w/w)**	52.10 ± 1.92*	66.24 ± 1.52*
**S** **ilver** **Sulphadiazine Ointment**	72.29 ± 2.35*	88.91 ± 2.59*

Values represents Mean ± S.D. (n = 6).

* p<0.01, when each treated group was compared with control and reference group.

The contents of antioxidants enzymes in skin lesions were significantly increased in 0.5% w/w PONG and 0.5% w/w LUT ointment-treated group as compared to control group (Table 5). No significant difference was observed in the group treated with 0.2% w/w PONG and 0.2% w/w LUT ointment. Slight increase in levels of SOD, CAT and GSH were found in groups treated with TPF-A and MAF-C ointments upto complete healing.

**Table 5:** Effects of ointment formulations from *M. annua* and *T. purpurea* on enzymatic and non-enzymatic level of tissues with burn wound in rats.

**Groups**	**Enzymatic and non-enzymatic assay**		
	**SOD (μg/50 mg tissue)**	**CAT (μmol/50 mg tissue)**	**GSH (μmol/50 mg tissue)**
**Control (Base)**	20.52 ± 1.05	25.24 ± 1.27	23.31 ± 0.85
**TPF-A Ointment (5% w/w)**	37.64 ± 1.32*	48.23 ± 1.86*	41.76 ± 1.34*
**MAF-C Ointment (5% w/w)**	39.12 ± 1.78*	46.17 ± 1.87*	45.69 ± 1.25*
**PONG Ointment (0.2% w/w)**	24.09 ± 0.94	26.34 ± 1.08	26.38 ± 0.76
**PONG Ointment (0.5% w/w)**	30.27 ± 1.21*	38.92 ± 1.27*	35.17 ± 1.28*
**LUT Ointment (0.2% w/w)**	23.61 ± 1.23	28.42 ± 0.94	28.20 ± 1.64
**LUT Ointment (0.5% w/w)**	28.28 ± 1.34*	36.16 ± 1.54*	37.64 ± 1.39*
**Silver Sulphadiazine Ointment**	38.28 ± 1.55*	50.38 ± 1.65*	46.88 ± 1.75*

Values represents Mean ± S.D. (n = 6).

* p<0.01, when each treated group was compared with control and reference group.

**Histopathological study**

**Figure 2:**
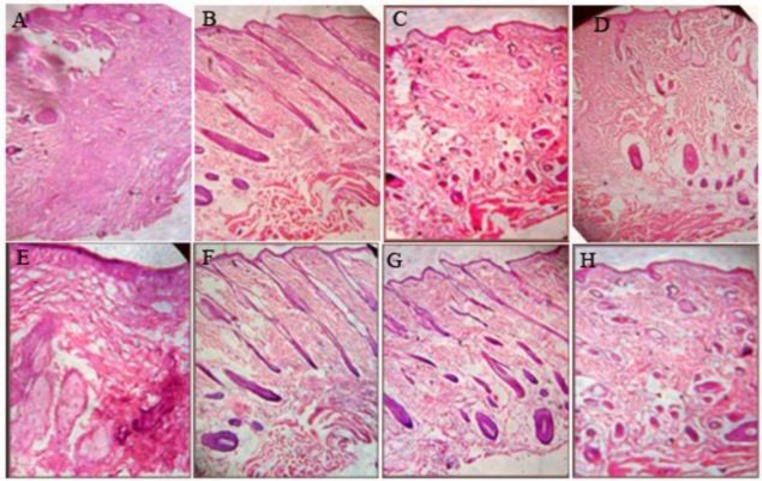
Photomicrograph of skin tissues after treatment with different fractions of *M. annua* and *T. purpurea* in dead-space wound. (A) Control; (B) TPF-A Ointment (5% w/w); (C) MAF-C Ointment (5% w/w); (D) PONG Ointment (0.2% w/w); (E) PONG Ointment (0.5% w/w); (F) LUT Ointment (0.2% w/w); (G) LUT Ointment (0.5% w/w); (H) Povidone Iodine ointment (5% w/w

**Figure 3:**
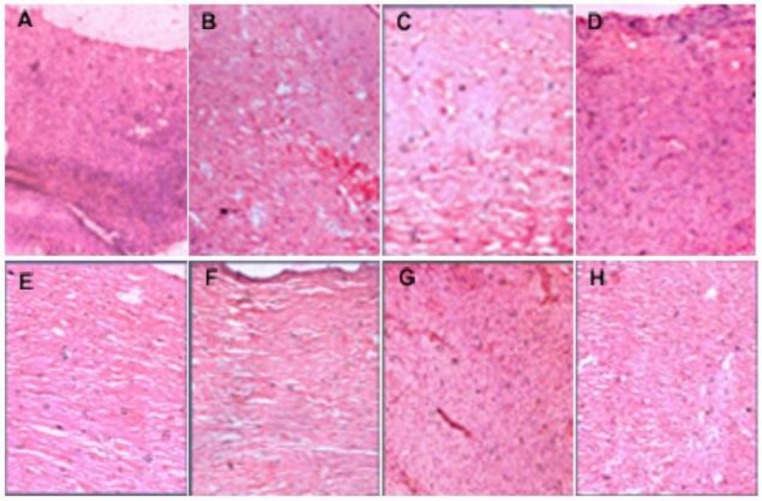
Photomicrograph of skin tissues after treatment with different fractions of *M. annua* and *T. purpurea* in burn wound. (A) Control; (B) TPF-A Ointment (5% w/w); (C) MAF-C Ointment (5% w/w); (D) PONG Ointment (0.2% w/w); (E) PONG Ointment (0.5% w/w); (F) LUT Ointment (0.2% w/w); (G) LUT Ointment (0.5% w/w); (H) Silver Sulphadiazine Ointment

In the group treated with MAF-C and reference ointment, wounds revealed the formation of angioblasts and fibroblasts throughout the tissue along with infiltration of neutrophils and microphages at few places on the 18^th^ day. The group treated with TPF-A showed the formation of fibrous connective tissue and blood capillaries (Figure 2). The arrangements of blood capillaries were parallel to that of fibrous tissue at some places and it was perpendicular to the fibrous tissue at other places. However, epidermal covering at few places was seen. The wound treated with 0.5% w/w PONG and LUT ointment had minute degrees of inflammation. The proliferation of collagen, fibroblasts cells and capillaries was observed with epidermal covering at the margins of the wounds (Figure 3). The skin lesions collected from control, and 0.2% w/w PONG and 0.2% LUT ointment groups had signs of chronic inflammation, with ulceration or progressing to ulceration (Figures 2 and 3). The tissue sections had acanthosis and dermal fibrosis, surface exudates, hyperkeratosis and epidermal inclusion cysts. It means lesions were in an intermediate stage of healing with residual changes (Figure 3). We concluded that after some time, the skin might be expected to return to nearly normal architecture in most or all cases.

## Discussion

Wound repair is a complex, integrated series of biochemical, cellular and physiological processes. Cutaneous wound repair is accompanied by a definite sequence of biological events starting with wound closure and progressing to repair and remodeling of damaged tissues (Rajan et al., 2004 ▶). 

The results of the present study were showed that ethyl acetate fraction of *T. purpurea *(TPF-A) and methanol-soluble fraction of *M. annua* (MAF-C) possess a definite healing action. TPF-A and MAF-C fractions containing pongamol and luteolin, respectively also have been found to promote wound healing at the concentration of 0.5% w/w. This is evidenced by a significant increase in hydroxyproline and protein content which was a reflection of increased collagen levels and was further supported by histopathological evidence. This indicated improved collagen maturation by increased cross-linking while an increase in dry granuloma weight indicated higher protein content. Increases in the level of antioxidants (SOD, CAT and GSH) were observed in granuloma tissues of dead-space, and burn wound model. These enzymes are known to quench the superoxide radical and thus prevent the damage of cells caused by free radicals. 

The role of collagen in wound healing begins immediately after the wound is formed and continues for months after it appears to be healed. Collagen is the predominant extracellular protein in the granulation tissue of wounds. Instantly following injury, there is an increase in the synthesis of collagen in wound area. Collagen plays a role in haemostasis and in providing strength and integrity to the wound matrix. It is also essential for re-epithelialization and cell–cell and cell–matrix interactions (Chithra et al., 1998 ▶; Raghow, 1994 ▶). As the wound heals, collagen molecules are synthesized and deposited at the wound site. These molecules become cross-linked to form fibers. The strength of the repaired wound tissue is a result of remodeling of collagen and formation of stable intra- and inter-molecular cross linking. These results may imply that flavonoids, as well as flavonoids containing fractions are able to increase collagen synthesis and possibly aid in formation of cross linkages as the collagen matures. 

Hydroxyproline is the major constituent of collagen and is found almost exclusively in collagen. The estimation of hydroxyproline is an acceptable method of biochemically evaluating the total collagen content of a sample and is also used as a marker of collagen synthesis. Glycoaminoglycans and proteoglycans are synthesized by fibroblasts in wound area. These substances form a hydrated gel-like ground substance (the provisional matrix) on which collagen is deposited (Agarwal et al., 2009 ▶). This was associated with a concomitant increase in total collagen content in treated groups after the induction of wound. This indicates replacement of granulation tissue in wound area by collagen. The protein content of granulation tissue is said to be an indication of protein synthesis and cell proliferation levels. If the protein contents of treated wounds are greater than the control group, it implies that the treatment stimulates cell proliferation. Thus, here, it is suggested that TPF-A, MAF-C and 0.5% w/w pongamol and luteolin appear to stimulate cell proliferation. Reduced glutathione is a potent free radical scavenger. Depletion of GSH results in enhanced lipid peroxidation. This can cause increased GSH consumption and can be related to the increase in the level of oxidized glutathione. Treatment with *T. purpurea* resulted in the elevation of GSH levels, which protect the cell membrane against oxidative damage by regulating the redox status of protein in the membrane. 

Superoxide dismutase is a protective antioxidant enzyme that catalyzes the dismutation of superoxide to yield hydrogen peroxide and oxygen. It scavenges superoxide ions which are produced as cellular by-products during oxidative stress (Al-Attar, 2011 ▶). CAT is involved in the elimination of H_2_O_2_. The functions of all enzymes are interconnected and a decrease in their activities results in the accumulation of lipid peroxides and increased oxidative stress in wounded rats (Liedias et al., 1998 ▶). Treatment with *M. annua* and *T. purpurea* increased the activity of these enzymes and thus may help to overcome free radicals production during severe wounds.

Immediately after thermal injury, polymorphonuclear neutrophil leukocytes (PMNs) invade the lesion, provoking the release of large amounts of oxygen free radicals and proteases in the interstitial fluid that cause endothelial cell and skin damage (Sabeh et al., 1998 ▶). The overproduction of free radicals and proteases impairs the production and release of growth factors such as the powerful angiogenic factor, vascular endothelial growth factor (Galeano et al., 2001 ▶). Indeed, healing is concomitant with an increasing release of angiogenic growth factors from macrophages and keratinocytes, and its impairment delays skin repair.

Flavonoids such as quercetin are believed to act as health-promoting substances as they have antioxidant and anti-inflammatory properties (Kleemann et al., 2011 ▶) and are reported to have important role in wound healing (Gomathi et al., 2003 ▶). In the inflammation phase, macrophages and neutrophils are attracted to the injured tissues that release inflammatory mediators, such as tumor necrosis factor alpha (TNF-a) and interleukin-1 (IL-1). Neutrophils contain high levels of destructive proteases and oxygen free radicals that are released into the wound area when cells die. This can cause extensive tissue damage and prolong the inflammatory phase. These free radicals are produced during oxidative stress, which causes lipid peroxidation, DNA breakage and scavenging enzymes inactivation. One of the major causes of delayed healing is the persistence of inflammation or an inadequate angiogenic response (Martin, 1996 ▶). It has been postulated that an anti-inflammatory response after cutaneous wound induction is a prerequisite for healing (Castangia et al., 2014 ▶). Potent antioxidant, anti-inflammatory agents such as quercetin can play an important role in restoring physiological conditions, allowing a significant improvement in wound healing. In conclusion, quercetin may be promising for wound healing, due to its ability to inhibit reactive oxygen species and tissue inflammation.

One approach in protecting from these harmful effects is to use antioxidants as photoprotectives. Naturally occurring phenolic compounds, flavonoids, and tannins have attracted attention as potent antioxidants (Svobodova et al., 2003 ▶). It was found that quercetin protected skin antioxidant systems, such as superoxide dismutase, glutathione peroxidase, glutathione reductase and catalase activities, against UVA (ultraviolet A) irradiating damage in rats (Inal et al., 2001 ▶). Selected flavonoids can directly scavenge superoxides and highly reactive oxygen derived radicals, which can inhibits low density lipoproteins (LDL) oxidation (Nijveldt et al., 2001 ▶). Flavonoids have been shown to increase collagen synthesis, decrease the degradation of soluble collagen, promote the cross-linking of collagen, accelerate the conversion of soluble collagen to insoluble collagen and inhibit the catabolism of soluble collagen. Clinically, collagen deposition in wound sites is the most important phase of healing. Declining free radical overproduction, facilitating oxygen diffusion, increasing lymphatic drainage and collagen synthesis were all together found to improve healing (Inan et al., 2006 ▶). 

In conclusion, our findings suggest that fractions of *M. annua* and *T. purpurea* have a potential benefit in enhancing the wound healing process. This effect may be due to the free radical scavenging property of flavonoids and plants. It is suggested that clinical studies with longer treatment periods are required to thoroughly evaluate the efficacy of plant extract ointment on skin wounds.

## Note

*Please cite this paper as: *

Lodhi S, Jain A, Jain AP, Pawar RS, Singhai AK. Effects of flavonoids from *Martynia annua* and *Tephrosia purpurea* on cutaneous wound healing. Avicenna J Phytomed, 2016; 6 (5): 578-591.

## Abbreviations

SOD, Superoxide dismutase; CAT, Catalse; GSH, Reduced glutathione; TCA, Trichloracetic acid; DTNB, Dithionitrobenzoic acid; PONG, Pongamol; LUT, Luteolin; TPF, *Tephrosea purpurea* fraction; MAF, *Martynia annua* fraction; TNF-a, Tumor necrosis factor alpha; IL-1, Interleukin-1.
